# Design and Application of New Low-Cost Instruments for Marine Environmental Research

**DOI:** 10.3390/s141223348

**Published:** 2014-12-05

**Authors:** Marco Marcelli, Viviana Piermattei, Alice Madonia, Umberto Mainardi

**Affiliations:** Laboratory of Experimental Oceanology and Marine Ecology, DEB—University of Tuscia, Molo Vespucci, Port of Civitavecchia, Civitavecchia 00053, Rome, Italy; E-Mails: marcomarcell@unitus.it (M.M.); alice_madonia@unitus.it (A.M.); info@maisoft.it (U.M.)

**Keywords:** low cost sensors, instrumentation development, chlorophyll *a* and Chromophoric Dissolved Organic Matter fluorescence

## Abstract

The development of low-cost instrumentation plays a key role in marine environmental studies and represents one of the most innovative aspects of current oceanographic research. These kinds of devices can be used for several applications, ranging from vertical profilers to stand-alone systems, and can be installed on different platforms (buoys, Voluntary Observing Ships, underwater vehicles, *etc.*). The availability of low-cost technologies enables the realization of extended observatory networks for the study of marine physical and biological processes through an integrated approach merging *in situ* observations, forecasting models and remotely sensed data. We present new low-cost sensors and probes developed to measure marine temperature, conductivity, chlorophyll *a* and Chromophoric Dissolved Organic Matter fluorescence, focusing on sensing strategies, general architecture, laboratory trials, *in situ* tests and comparison with standard instruments. Furthermore, we report the expendable (*New T-FLaP*), vertical profiler (*T-FLaPpro*) and stand-alone (*Spectra*) applications of these technological developments that were tested during several oceanographic surveys in the Mediterranean Sea.

## 1. Introduction

Marine ecosystem monitoring is nowadays a main concern in the worldwide scientific community [1]. A significant amount of effort has been invested modelling marine dynamics and processes, and there are exhaustive scientific programs and technological developments, e.g., Argo floats [2] and Expendable BathyThermograph (XBTs) probes [3], to measure physical and biological variables.

Ocean sciences are strictly dependent on the development of new sensors and platforms, providing multi-disciplinary datasets, advanced sampling strategies and the expansion of temporal and spatial coverage of our measuring capacity of natural phenomena. Ocean measurement provides a long story of improvement in instrumentation capacity, from the accuracy, precision and resolution, until quality control and precision manufacture [4]. Technological advances in oceanographic measurement capabilities have produced new ways to observe and monitor the ocean that improve operational and forecasting oceanography. Operational oceanography currently represents a part of the ocean sciences that provides high-quality observational data and models for research and practical applications [5,6], but critically depends on the near-real-time availability of a large amount of *in situ* data collected with sufficiently dense spatial and temporal sampling. This issue directly influences the robustness of ocean forecasting models and remote sensing observations through data assimilation and validation processes. Extended observatory networks represent an important new field in the study of global phenomena, through the development of cheap, small and integrated smart sensors [7].

Along these lines, the use of low-cost instrumentation from ships of opportunity, promoted by international research programs, is gaining more and more attention. This instrumentation makes it possible to reduce the costs of oceanographic surveys and, at the same time, to improve data spatial density and coverage and collect relevant data about studied phenomena [8].

Furthermore, the new emerging Ecosystem-based Management method proposed by the Marine Strategy Framework Directive (MSFD) [9] (2008/56/EC) requires an integrated approach composed of *in situ* observations, forecasting models and remotely sensed data for the monitoring and assessment of the environmental status of marine ecosystems. Although the existing technology allows to deploy sensors and probes on buoys, mooring and many other kind of platforms [10], the MSFD objectives cannot be achieved with current marine measurement technologies, which are too expensive for extensive utilization.

The Ship Of Opportunity Programme and the Voluntary Observing System represent the most important world initiatives that largely rely on (XBTs) [11,12]. Since the 1960s, XBTs have been successfully adopted by oceanographers as an easy way to collect temperature profiles using commercial ships. Even now, XBT remains the most effective method for low-cost and simple acquisitions of temperature profiles, although the quality of these data must be checked [13].

Despite the development and extensive use of this kind of sensors to provide near real-time analysis of the ocean temperature, gaps remain in the estimation of certain biological variables (e.g., phytoplankton biomass, Chromophoric Dissolved Organic Matter). Observation and study of the distribution of biological variables plays a key role, particularly at mid-low latitudes, where a deep observation of the water column is needed because of the typical distribution of phytoplankton biomass [14]. Chlorophyll a (Chla) fluorometry is the principal method to study phytoplankton biomass in the ocean. However, the high costs of commercially instrumentation have made it unavailable for extensive use [15]. Dissolved organic matter (DOM) in the oceans is one of the largest reserves of reactive organic carbon on Earth [16] controlling the concentration of CO_2_ in the atmosphere and affecting global climate change [17]. CDOM, also known as gelbstoff or yellow substance, is that component of DOM that absorbs light over a broad range of visible and UV wavelenghts, having a strong impact on the availability and spectral quality of light in photosynthesis [18]. The emission fluorescence of CDOM covers a wide spectral range at blue-green wavelengths, allowing to estimate its concentration in natural waters. Furthermore, together with phytoplankton, CDOM is one of the most optically active components of the oceans [19,20], representing a key parameter in primary production estimates with bio-optical models. A significant effort has been undertaken recently to develop a system that enables both high-performance measurements of physical and biological variables and high flexibility and low costs of the production compared with traditional instruments. Our aim was to realize a technology that could be adapted for use in different monitoring systems to provide continuous data collection [21].

In this paper, we present a new low-cost system for marine temperature, conductivity, Chla and CDOM measurement developed by the University of Tuscia Laboratory of Experimental Oceanology and Marine Ecology, the last upgrades and *in situ* tests.

## 2. Low-Cost Technology

With the aim of adding biological profiling measurements to the physical measurements performed by commercial XBTs, in 2003 Temperature-Fluorescence Launchable Probe (*T-FLaP*) technology was developed and the first 30 prototypes were tested in the frame of the Mediterranean Forecasting System: Toward Environmental Prediction (MFS-TEP) project [5,11–24]. *T-FLaP* is an expendable fluorometer designed to obtain vertical temperature and fluorescence of Chla profiles along the water column by moving ships. The probes provided real-time temperature and Chla fluorescence profiles up to a depth of 500 m with an accuracy of 0.1 °C and 0.1 mg/m^3^, respectively.

*T-FLaP* technology has recently been refined and improved. The new advances can be summarized in the following points:

- replacement of Chla fluorescence and temperature sensors with more sensitive devices (temperature accuracy: 0.01 °C; Chla accuracy: 0.01 mg/m^3^);
- integration of a miniature 500 dBar pressure transducer, of a triaxial accelerometer and a gyroscope (optional) for the study of the dynamic behavior of the probes following the release;
- development of new sensors for the measurement of conductivity and CDOM fluorescence;
- improvement of miniaturized electronic devices;
- realization of a modular measuring cell;
- reduction of light-reflection phenomena inside the measuring cell by means of an anodic oxidation treatment with a matte black covering on the mechanical components.

Thanks to these innovations, it has been possible to improve the expendable probe and develop two additional new applications usable and adaptable to different scientific and operational needs: the recoverable vertical profiler *T-FLaPpro*, for use as a Conductivity Temperature Depth (CTD) probe from research vessels, and the stand-alone system *Spectra*, working as a mini-ferrybox for surface mapping of the bio-physical properties of seawater.

### 2.1. Main Goals

Ease of use, low cost and modularity are the basic principles of this technology, which is ideal for deployment from voluntary boats, even by non-specialized operators. A significant effort was dedicated to the selection of low-cost components, in order to realize a technology that could be implemented in extended monitoring systems. Despite the low-cost philosophy, all of the sensors were made with the aim of identifying, with good accuracy, the physical structures and biological phenomena of the oceans (thermocline, pycnocline, Deep Chlorophyll Maximum (DCM), *etc.*). The modularity of the measuring cell allows us to combine the sensors depending on specific needs: the system has been designed to be flexible and customizable enough to address heterogeneous operative demands and oceanographic platform installation. Furthermore, much effort has been expended to ensure the miniaturization of electronic components and a reduction in packaging, which allows us to increase the internal volume available for the inclusion of new features in the future.

### 2.2. General Architecture

**Figure 1.**
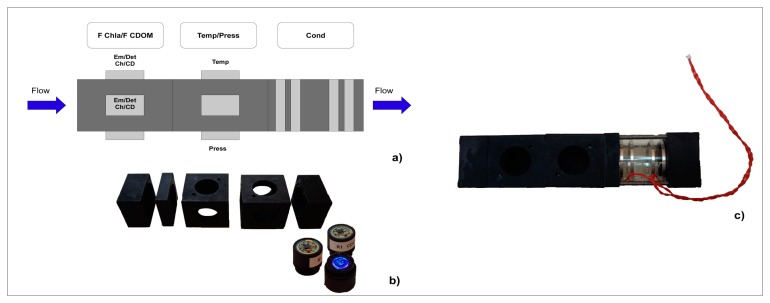
(**a**) Possible combinations of the sensors inside the measurement cell; (**b**) Modules of the measurement cells; (**c**) A prototype of the measurement cell.

The new expendable, vertical profiler and stand-alone instruments present different external cases with technical characteristics adapted to their operative use (submersible, waterproof, expendable *etc.*). Nevertheless, they share a standard internal measuring cell (Figure 1). The measuring cell is a flow-through tubular cell where the water flows. As discussed above, the measuring cell has a modular structure. The modules are aluminium cubes 40 × 40 × 40 cm, placed in sequence and perforated to allow the water to flow. The design of the measuring cell has been studied in order to ensure that the sensors are in direct contact with the volume of water passing through the probe.

Each of the modules can accommodate up to three cylindrical, interchangeable supports containing all of the possible elements for physical and biological sensing. Each probe can house the following configurations:

- temperature sensor;
- pressure sensor;
- excitation/detection devices of the Chla fluorometer;
- excitation/detection devices of the CDOM fluorometer.

**Figure 2.**
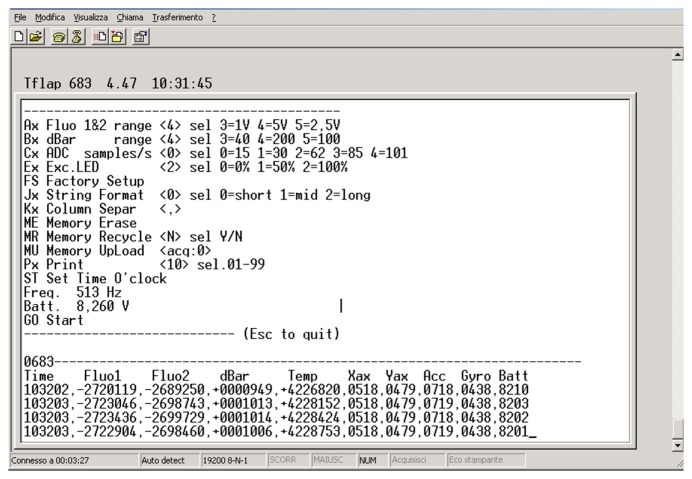
Terminal screen showing the different instruments and sensor configurations and controls.

The probes can be easily taken out of the cell to facilitate cleaning the optics or replacement operations in the case of biofouling. All of the mechanical components are completely anodized and covered with a matte black deposit to reduce scattered light emitted within the cell. Batteries and electronic boards are placed on the external part of the measuring cell. A specific battery package makes the instrument totally autonomous, depending on the operative time required for each application. The electronic boards are placed around the measuring cell, allowing for the management of the power supply and the modulation of light sources, and the control of the the sensor signal, the signal conditioning, the digital conversion functions and the data transmission. The firmware of the instrument allows it to communicate in real time with a user through a remote terminal, in order to switch on and configure the instrument with the best measurement conditions. In particular, the program makes it possible to set and control a series of configurations and information, including the operative ranges of the parameters, LED excitation, battery and memory status. An example of the terminal screen before and during acquisition is illustrated in Figure 2.

## 3. Sensors

### 3.1. Temperature and Pressure Sensors

The temperature microsensor consists of a sensitive bulb (1.5 mm in diameter), located on the top of a quartz stem 10 mm in length. The sensitive part is a thermistor inserted into the glass bulb. Given its minimum dimensions, this sensor has the sensitivity to detect temperature variations of 0.01 °C with a response time of 0.05 ms [22]. Depth is measured through a submersible pressure transducer (**KELLER AG für Druckmesstechnik, Winterthur, Switzerland**).

### 3.2. Chlorophyll a Fluorometer

**Figure 3.**
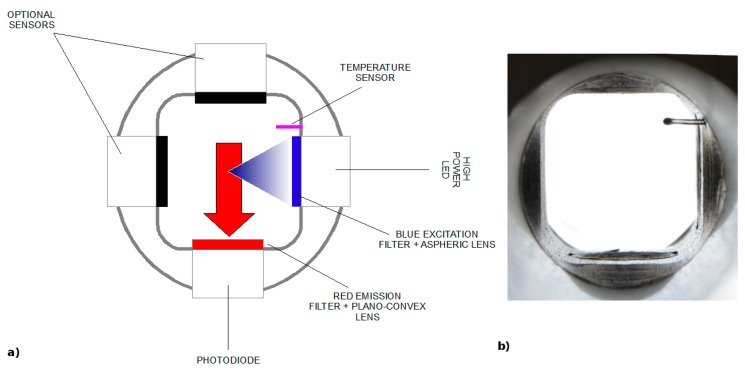
(**a**) Scheme and (**b**) an internal view of the new fluorimetric cell.

**Figure 4.**
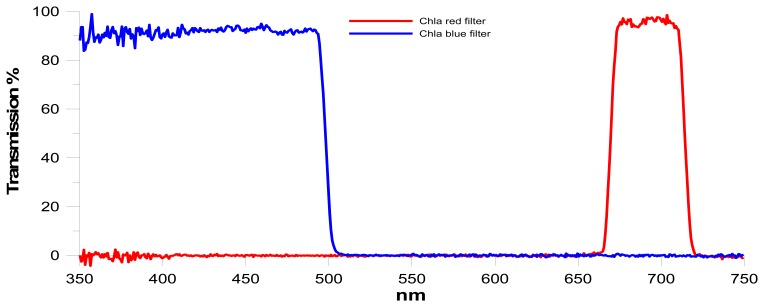
Transmission spectra of the optical filters in the excitation (**Blue Curve**) and in the detection (**Red Curve**) modules obtained by an Ocean Optics QE65000 spectrometer.

The Chla fluorometer comprises an emitter and a detector positioned orthogonally to each other, in order to reduce the amount of excitation light scattered to the detector (Figure 3). The emission module is equipped with a blue high-power LED light source (430–470 nm), an optical short-pass filter and an aspheric lens. Blue light excites Chla pigments of phytoplankton cells passing through the measuring cell. The detection module, composed of a red optical band-pass filter, a plano-convex lens and a silicone photodiode, receives the fluorescent signal produced by the excited cells, and selects, focuses and amplifies the red peak between 682 and 685 nm, as shown in Figure 4. The insertion in the emission/detection modules of optical windows with high transmission to incident radiation constitutes a useful tool for the maintenance of the sensors, particularly for making sure that the optics of the instrument do not become biofouled.

### 3.3. CDOM Fluorometer

**Figure 5.**
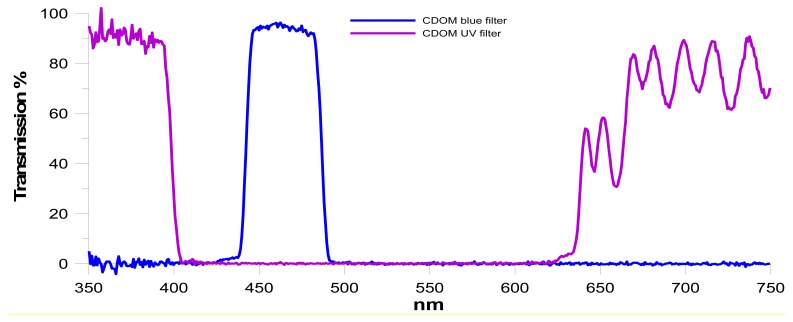
Transmission spectra of CDOM optical filters in the excitation (**Violet Curve**) and detection (**Blue Curve)** modules obtained by an Ocean Optics QE65000 spectrometer.

The CDOM fluorometer has the same structure as the Chla fluorometer, with orthogonal emission and detection modules, except for using LED and optical filter wavelengths. The excitation source produces ultraviolet radiation (370 nm) and the receiving device selects the CDOM fluorescent signal (400–500 nm), transmitting it to the photosensitive element, as shown in Figure 5.

### 3.4. Conductivity Sensor

The conductivity sensor consists of a specific cylindrical module containing four platinum rings placed on the flow line. The outer rings generate an electric current and the inner rings measure the potential difference that is determined by the induced current. Using an electronic control circuit, the potential difference is kept constant in time. The current generated by the outer rings is controlled and measured by the electric circuit of the reaction in such a way that it increases and decreases proportionally, depending on variations in the conductivity.

### 3.5. Calibration System

**Figure 6.**
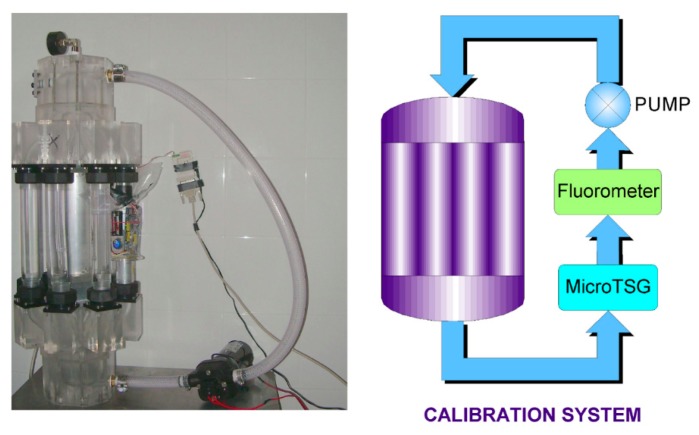
The calibration system.

The calibration procedure is a very important step in the development of new technologies. In order to convert the electrical outputs of each sensor to physical values, we designed and realized an automatic calibration system that allows us to calibrate the instruments in the laboratory, before their use. The calibration system is a closed hydraulic circuit composed of a calibration chamber that can house eight probes at a time, a circulation heat exchanger, a pump for the water flow and the control sensors (Figure 6).

The calibration chamber has a modular construction including a manifold, flow distributors and pipelines. All components were realized by subjecting two plexiglass blocks to laser manufacturing to produce the smoothest possible finish and to ensure flow uniformity and a minimum loss of pressure. The manifold was produced to simultaneously distribute both water and pressure inside the system.

Above the upper manifold, a Millipore two-way vacuum valve makes it possible to inject the specific standard solutions for the calibration of the Chla and CDOM fluorometers (phytoplankton cultures or quinine sulphate dissolved in 0.05 M solutions of H_2_SO_4_) into the system [25]. The water flow is regulated by a high-capacity, low-velocity pump located adjacent to the control sensors. The outflow pump goes through the temperature conditioning system and upper manifold via the eight probes and is collected in the lower manifold before the water flows through the control sensors and ultimately returns to the pump. The flow is closed and hermetic.

**Figure 7.**
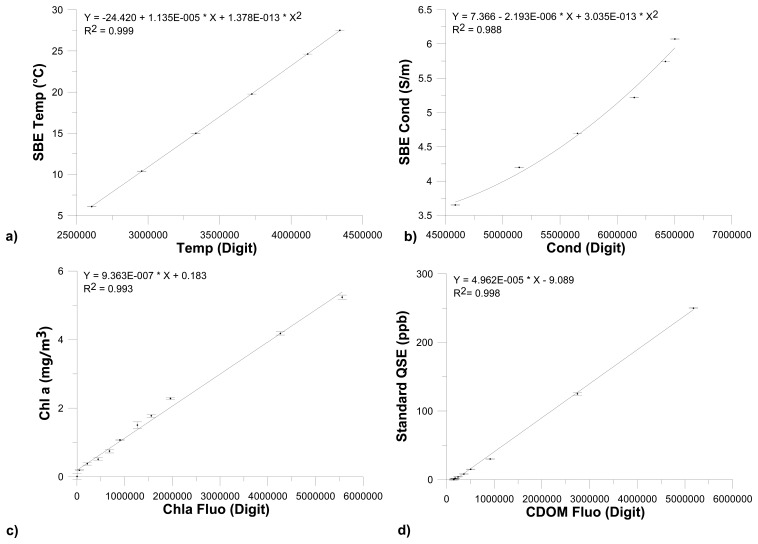
Examples of (**a**) temperature; (**b**) conductivity; (**c**) Chla fluorescence and (**d**) CDOM fluorescence calibration curves.

The control sensors system forms a collateral circuit. Temperature and conductivity were measured by a MicroThermosalinograph (MicroTSG; SBE 45 Sea-Bird Electronics, Bellevue, WA, USA; temperature resolution: 0.0001 °C; conductivity resolution: 0.0001 mS/cm) and fluorescence was controlled by a flow analysis system (FIAlab Instruments by Ocean Optics, Bellevue, WA, USA; spectral range 310–750 nm; photomultiplier based for ultra-low fluorescence). In Figure 7, we show an example of the calibration curves of temperature, conductivity, Chla and CDOM fluorescence sensors. Concentration abundances of phytoplankton solutions used for the calibration of Chla fluorometers were also analyzed using the standard spectrophotometric method reported in [26,27].

## 4. Prototype Applications

### 4.1. New T-FLaP: The Expendable Probe

As discussed previously, the basic application of this technology has been the expendable one. Improvements to the first *T-FLaP* started with the Adriatic Sea Integrated Coastal Areas and River Basin Management System Pilot Project in 2008 [28].

The mechanical design of the prototype meets different requirements as: a good falling velocity, which allows both the coil unroll and the adequate number of acquisitions; the modularity of the cell that, unlike earlier prototypes, in the new *T-FLaP* allows to change the configuration of the sensors. Furthermore the probe is equipped by a specific tail designed and realized to spin the probe during a fall; the rotation allows the coil to correctly unroll itself.

**Figure 8.**
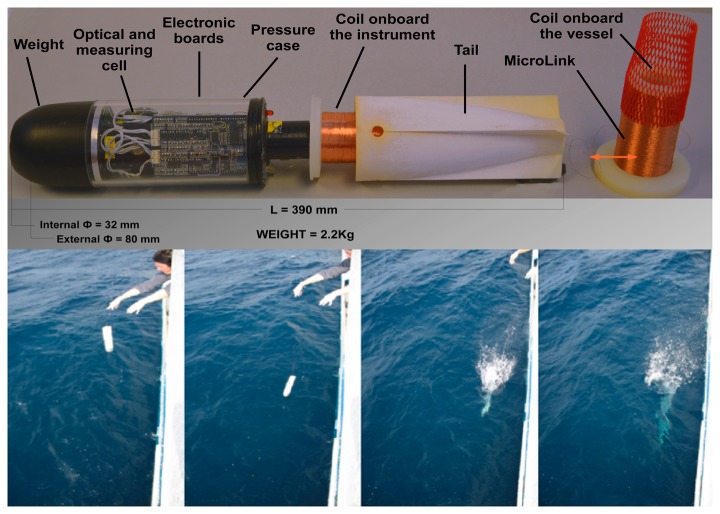
A new *T-FLaP* prototype with a transparent case for electronic visualization and the launch during ADR0208 survey.

As shown in Figure 8, digital data transmission is assured by twin copper wires wrapped on two reels: one in the probe tail and the other onboard the ship. This setup ensures a connection with the computer until the signal interrupted by a broken wire. Data transmission is provided by an RS-485 serial interface and the communication rate is 19,200 baud, no parity check, one bit stop and no flow control. Data can be acquired on a pc through a serial converter RS-485/232 and a terminal as Windows^®^ HyperTerminal, following the information transmitted by the connected device.

#### Field Tests

The ADR0208 Oceanographic Cruise coordinated by the Institute of Atmospheric Sciences and Climate of the Italian National Research Council (SAC-CNR) departed on 17 October 2008 from the Port of Bari and ended on 28 October of the same month. The research area consisted of the Southern Adriatic Sea, the Montenegro and Albanian coastal zone and the Boka Kotorska Bay.

The Adriatic Sea is a high productivity area and estimates based on the Coastal Zone Color Scanner (CZCS) confirm that the Adriatic Sea has the highest pigment biomass and primary production of all Mediterranean sub-basins [29,30].

**Figure 9.**
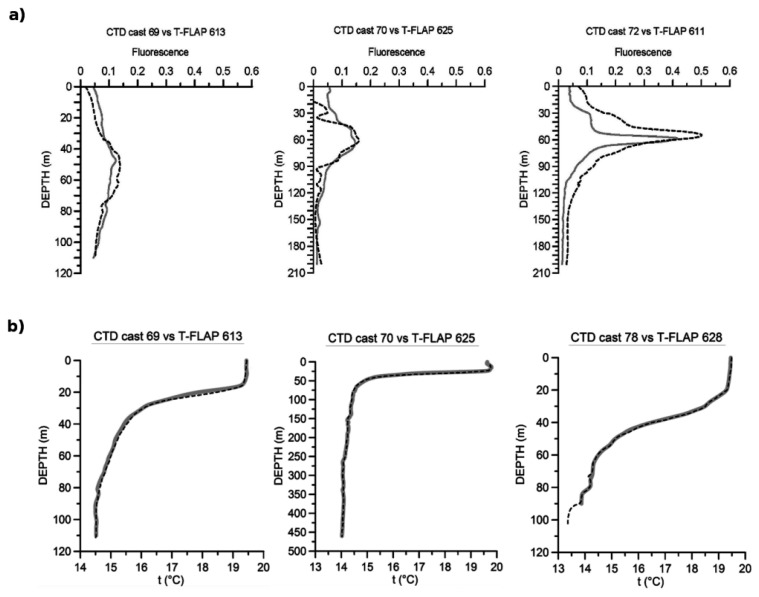
(**a**) Comparison between *T-FLaP* (dashed dark line) and Chelsea Aquatraka III (solid grey line) fluorescence profiles and (**b**) Comparison between *T-FLaP* (dashed dark line) and SBE911 (solid grey line) temperature profiles.

This dynamic area was chosen to test the sensors' responses at different trophic conditions. Five new *T-FLaPs* were launched along the Dubrovnik-Bari transect at the end of the survey. In each *T-FLaP* station, standard CTD casts and water sampling were performed using a standard SBE 911 CTD probe with a Chelsea Aquatraka III fluorometer and a rosette sampling system. Since we did not use *T-FLaP* from a moving ship, we were able to compare the *T-FLaP* fluorescence measurements with *in situ* Chla, as shown in Figure 9.

In the first 5–10 m of the water column, depending on the lighting conditions, the *T-FLaP* fluorometer response can be unreliable due to the saturation of the photodiode; the response becomes consistent at deeper depths. The discrepancy between the fluorescence profiles at cast 72 is due to the greater amount of time elapsed (about one hour) among the lowering of the two instruments. The vertical temperature profiles show how *T-FLaP* can resolve the structures detected by the CTD with good resolution (Figure 9b). Although the *T-FLaP* temperature sensor has an accuracy of 0.01 °C with a dynamic response time of 1.5 ms/°C, the sensor performances are obviously lower than that of SBE [22]. The main differences between the two probe profiles are more evident in the zone of rapid temperature changes. In fact, even if the accuracy and the response time of the sensor are sufficient for expendable use, the relation between the falling velocity and data transmission rate is the most important operational aspect, with the *T-FLaP* now capable of producing data information at every meter of depth.

### 4.2. T-FLaPpro: The Vertical Profiler

**Figure 10.**
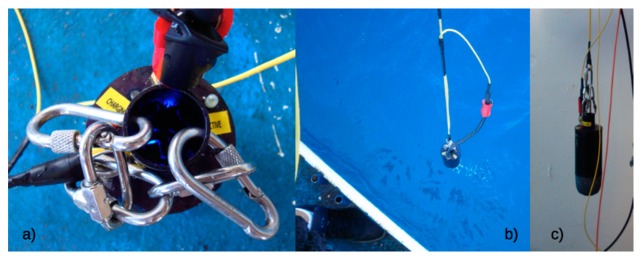
The *T-FLaPpro*: (**a**) blue LEDs inside the measure cell; (**b)** the probe during the launching; (**c**) a wide-angle view of the probe.

Based on previous applications, a non-expendable vertical profiler (*T-FLaPpro*) was realized (Figure 10), in order to create a small and low-cost multiparametric probe that can be easily used onboard coastal and smaller vessels. The largest differences are found between the transmission system and the launching methods. In particular, the probe was provided with an underwater connector and a cable developed *ad hoc* for this application, which allows for real-time data transmission and visualization.

#### Field Tests

A series of tests were carried out during the phytoplankton bloom period in order to observe the DCM structure. For this purpose, a survey was conducted off the shore of Civitavecchia (Tyrrhenian Sea, Latium, Italy) coastal area on 16 April 2013.

**Figure 11.**
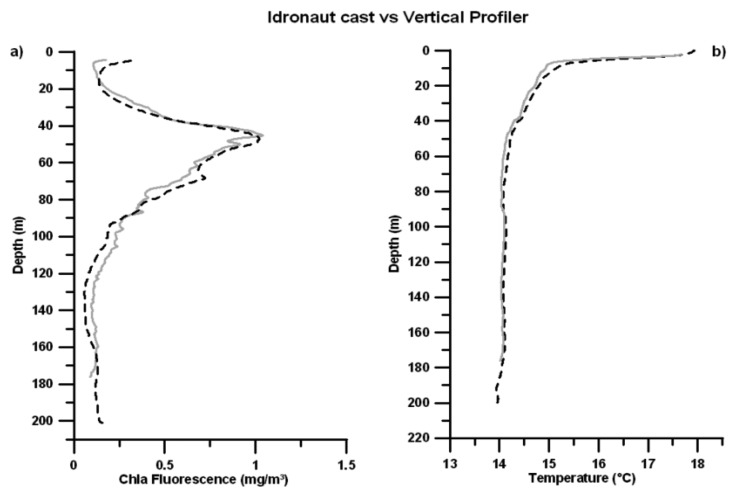
(**a**) Comparison between *T-FLaPpro* (dashed dark line) and Seapoint (solid grey line) fluorescence profiles and (**b**) comparison between *T-FLaPpro* (dashed dark line) and IDRONAUT OCEAN SEVEN 316Plus (solid grey line) temperature profiles.

A vertical profile along the water column was performed by *T-FLaPpro* and, at the same time, by the IDRONAUT OCEAN SEVEN 316Plus probe, which was equipped with a temperature sensor and the Seapoint fluorometer. Therefore, it was possible to compare the profiles, as shown in Figure 11, and to analyze the capability and sensitivity of *T-FLaPpro* to detect the DCM structure.

An analysis of the distribution of variables along the water column shows an almost-complete overlap between the profiles acquired with the *TFLaPpro* and the probe IDRONAUT OCEAN SEVEN 316Plus. In particular the Chla fluorescence profile shows an intense DCM, reaching concentrations of 1 mg/m^3^.

### 4.3. Spectra: The Stand-Alone System

The last prototype, derived from the original technology, is an in-line measuring system, which provides continuous real-time information about the physical and biological states of the surface waters through which the vessel passes. It was developed to be used onboard both coastal, smaller vessels and ships of opportunity such as ferries and commercial ships. The philosophy that inspired this instrument is therefore that of Ferrybox, but with miniaturization of components and a considerable reduction in costs.

**Figure 12.**
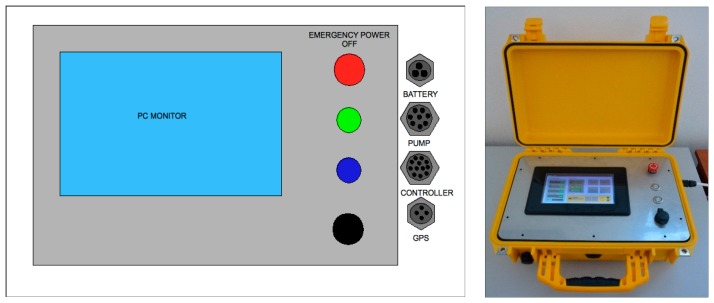
The electronic unit: the scheme (**Left**) and the realized prototype (**Right**).

**Figure 13.**
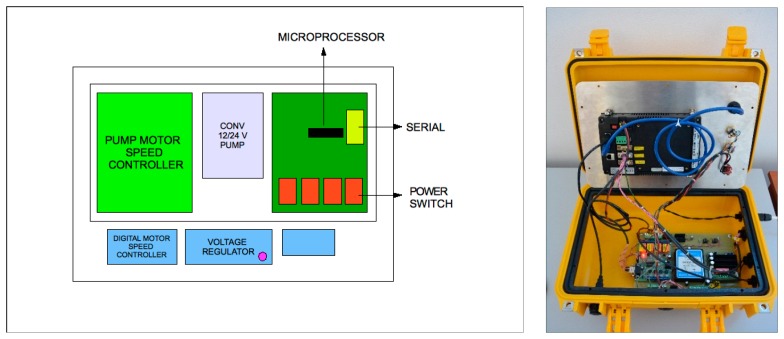
The control unit: The scheme (**Left**) and the realized prototype (**Right**).

**Figure 14.**
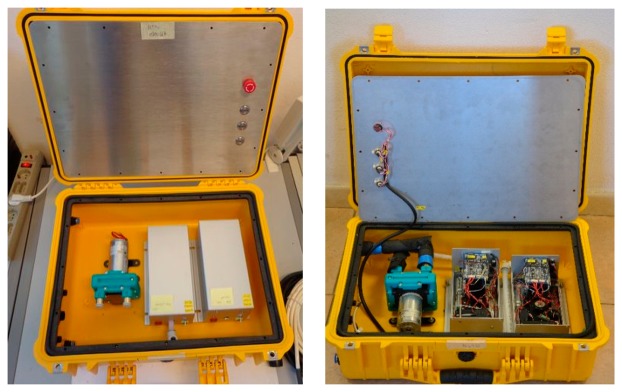
The hydraulic and measuring unit: The measuring unit with the area closed (**Left**) and the measuring unit with the area open (**Right**).

This system, called *Spectra*, is composed of three main modules:

- The electronic unit dedicated to the data acquisition, transmission and storage (Figure 12);
- The control unit (Figure 13);
- The hydraulic and measuring unit (Figure 14).

The first unit, assembled in a standard *Peli* Protector Case (L40 × L32 × H17), includes the computer, with a waterproof touch-screen display to manage the acquisition and visualization of data via customized software, and the GPS, essential for measuring position.

The control unit, placed under the electronic unit, manages the turning on and off of the devices and the signals of all the payloads; the control unit also controls the pump velocity. The hydraulic and measuring unit, assembled in a standard *Peli* Protector Case (L50 × L42 × H22), houses the flow through the system composed of the diaphragm pump and the two modular measurement cells (L22 × L10.5 × H10.5), equipped with temperature, conductivity, Chla and CDOM fluorescence sensors.

#### Field Tests

**Figure 15.**
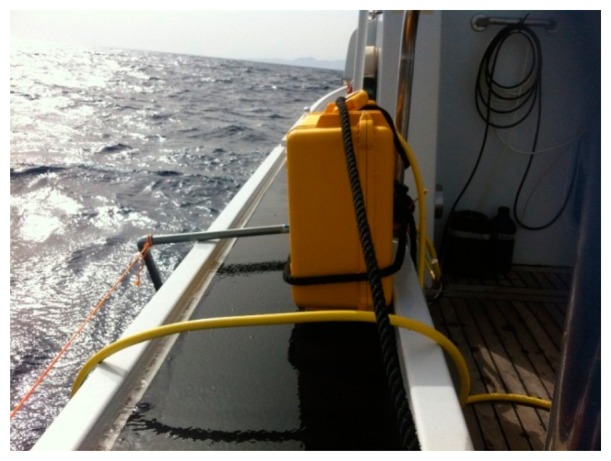
Spectra system installed on board a coastal vessel.

The field tests of the stand-alone application were performed along the Civitavecchia (Central Tyrrhenian Sea, Italy) coast on 4 February 2014. Spectra was fixed on the poop deck of a small coastal vessel and equipped with an input/output pipe system able to transfer water from the sea surface to the internal measuring cells (Figure 15). The stand-alone application was extremely versatile and the integrated pumping system allowed for installation on different types of vessels.

**Figure 16.**
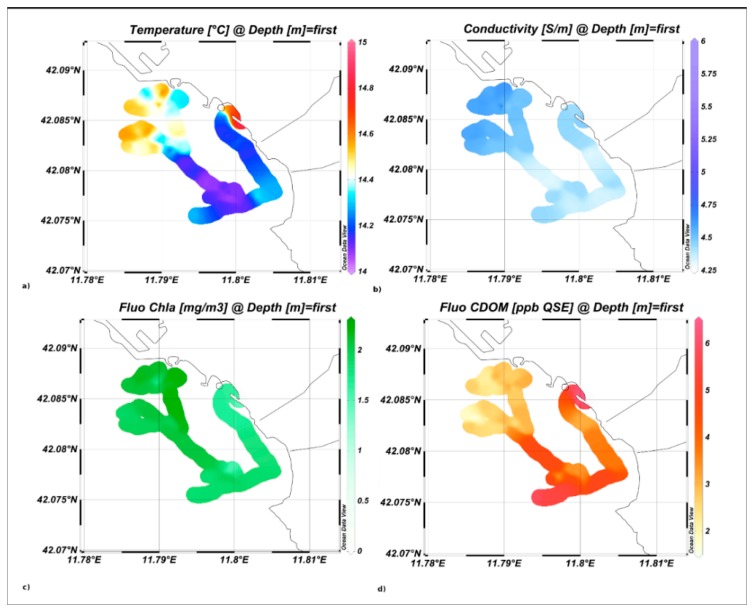
*In situ Spectra* acquisitions: (**a**) temperature; (**b**) conductivity; (**c**) Chla and (**d**) CDOM fluorescence isosurface maps.

This area was chosen because of the presence of urban discharges and effluents that can be characterized by a high variability in both physical and biological parameters. Temperature and conductivity isosurface maps, shown in Figure 16, exhibit lower values in the southern area, characterized by the presence of the Scarpatosta River.

Chla and CDOM fluorescence results are inversely correlated, with lower Chla abundances and higher CDOM levels in the southern study area, indicating the relevant intake of urban water masses, strongly connected to the heavy precipitations that occurred in the previous days.

## 5. Conclusions and Future Directions

We have presented an advanced technology for marine bio-physical measurements. The sensors and the probes that we have developed allow integrated and distributed low-cost marine environmental monitoring. The cost of the prototypes (not engineered) ranges from 1200 Euros for the expendable application (a corresponding probe is not available on the market), 2500 Euros for the *T-FLaPpro* up to 5000 Euros for the Spectra, far below the cost of the equipment that is currently on the market. The typical investment costs of this kind of instrumentations is much higher: a standard oceanographic fluorometer cost approximately between 1900 and 9000 Euros, while a FerryBox starts at 25,000 Euros. Despite the lower prototype costs, the measurement accuracy of the different variables is very high and comparable with standard commercial instruments. A comparison between the data acquired by the new technological developments and traditional probes yields strong consistency, satisfying the resolution requirements for the description of the physical and biological structures with good accuracy. The expendable and the vertical profiler were shown to be capable of identifying the distribution of phytoplankton populations along the water column both in eutrophic and oligotrophic marine waters. The *in situ* application of the Spectra prototype confirmed the validity of the measurement system developed for temperature, conductivity, Chla and CDOM fluorescence acquisition on the surface layer of the sea, which can be a useful tool for calibrating and validating remotely sensed data and mathematical models.

These technological solutions were specially designed to be used onboard ships of opportunity and for the implementation of oceanographic monitoring networks, in order to improve the quality of operational oceanography products. The availability of extended datasets is fundamental to our understanding and forecasting of physical and biological marine processes.

In conclusion, we have confirmed progress in technological development and provided important research directions for future work. In particular we are working on different aspects: the development of new sensors (e.g., backscatter) and the integration of this technology on other platforms (e.g., AUVs, buoys and fixed stations). Both these activities are now under testing and we are planning different experimental surveys. The improvement of this technology and its integration in different oceanographic platforms will be an important progress toward a cost-effective extended monitoring network.
